# Prediction for Respiratory Failure in Ischemic Stroke Patients Admitted to ICU: A Retrospective Analysis Based on MIMIC‐IV Database

**DOI:** 10.1002/brb3.71146

**Published:** 2025-12-22

**Authors:** Zhenjun Liu, Luolan Gui, Qian Zhao, Yi Li

**Affiliations:** ^1^ Department of Critical Care Medicine, Sichuan Cancer Center, School of Medicine, Sichuan Cancer Hospital and Institute University of Electronic Science and Technology of China Chengdu Sichuan Province China; ^2^ Laboratory of Clinical Proteomics and Metabolomics Institutes For Systems Genetics Frontiers Science Center For Disease‐related Molecular Network National Clinical Research Center for Geriatrics West China Hospital Sichuan University Chengdu Sichuan Province China

**Keywords:** cerebral infarction, Cox proportional hazard model, MIMIC database, MSCM, nomogram

## Abstract

**Introduction:**

There remains a lack of studies evaluating the risk of respiratory failure in intensive care unit (ICU)‐admitted ischemic stroke (IS) patients. We aim to develop a nomogram for the prediction of respiratory failure in those patients and the identification of the patients with high risk of respiratory failure, to facilitate early intervention.

**Methods:**

The medical data of IS patients in the Medical Information Mart for Intensive Care (MIMIC)‐IV database were extracted. Variables were selected using Cox stepwise regression, and variables with statistical significance were finally included in the nomogram. The marginal structural Cox model (MSCM) was to adjust for baseline and time varying confounding factors. The calibration curve and Receiver operating characteristic curve (ROC) were applied to assess the performance of the model.

**Results:**

External validation using IS patient data from the eICU collaborative ersearch database (eICU‐CRD). A total of 3462 eligible patients (2424 in the training set and 1038 in the validation set) were included. The following variables were finally included in the model: infarction location, atrial fibrillation, A alkaline phosphatase (ALP), anion gap (AG), lactic dehydrogenase (LDH), and Na^2+^ concentration. The direction of the hazard ratios (HR) of the variables in the model is consistent with the MSCM results. The area under the ROC curve (AUC‐ROC) of respiratory failure occurring between 1 and 7 days after ICU admission was 0.839 and 0.760 in the training set, 0.839 and 0.769 in the validation set, and 0.687 and 0.733 in the eICU set, respectively. The calibration curve showed acceptable consistency, indicating the model was of satisfactory performance.

**Conclusion:**

We have developed a nomogram model for the prediction of respiratory failure in IS patients admitted to the ICU, validated using external data. The model could perform effective prediction and thus provide more information for clinicians.

## Graphical abstract

Development and validation of a multicenter data‐based risk prediction nomogram model for respiratory failure in ICU patients with ischemic stroke.

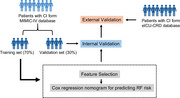

## 1 Introduction

IS is the second leading cause of death and disability on a global scale, and its morbidity and mortality remain increased due to the aging of the population (Virani et al. 2020, Donnan et al. 2008). It should be noted that acute IS is a heterogeneous disease. Thoroughly examining patient variability and distinguishing between different stroke subtypes is of great significance for studying its risk factors, severity, and prognosis (Gasull and Arboix 2022). IS could induce a series of neuropath logical events, beginning with energy‐metabolism dysfunction and followed by ionic imbalance, glutamic acid release, calcium channel dysfunction, free radical generation, cellular membrane destruction, inflammation, and cell death (Dirnagl et al. 1999). IS has been demonstrated to have adverse effects on the respiratory system, and the severity of respiratory disturbance depends on the location and extent of neuronal injury (Stewart 1999), which could lead to critical complications such as respiratory control dysfunction, sleep‐related respiration disorder, swallowing dysfunction, aspiration pneumonia, and neurogenic pulmonary edema (Rochester and Mohsenin 2002). Moreover, studies have demonstrated that IS could induce lung injury and reduce the phagocytosis of alveolar macrophages in rats (Samary et al. 2018). These complications significantly impact the survival and prognosis of IS patients, while most of them could be treated via early intervention. Respiratory disturbance might not be the primary concern in the early management of IS, but improvement of respiratory function is a vital part of the subsequent treatment.

However, there is a lack of studies focusing on the identification of predictors for severe respiratory disturbance in IS patients. IS patients who need intensive care are more critical (Duan et al. 2021), with a higher incidence of respiratory complications. The aim of this study was to construct a nomogram to predict the risk of respiratory failure in IS patients admitted to the ICU and to validate it externally. In order to identify high‐risk patients and intervene early to improve their prognosis.

## 2 Material and Methods

### 2.1 Data Source

Data of IS patients in the Medical Information Mart for Intensive Care (MIMIC)‐IV database (Johnson et al. 2023) (ver.2.0, available from https://physionet.org/content/mimiciv/2.0/) and the eICU database (Pollard et al. 2018) (ver.2.0, available from: https://physionet.org/content/eicu‐crd/2.0/) were extracted using PostgreSQL (ver.14, https://www.postgresql.org/). The exclusion criteria were:

1. IS patients who had not been admitted to ICU.
2. Occurrence of respiratory failure before ICU admission (Arterial partial pressure of oxygen (PaO2) <60 mmHg or PaO2/FiO2 ≤ 300 mmHg under oxygenation).
3. Patients receiving mechanical ventilation.
4. Patients with missing data.

Collected data form MIMIC‐IV database included demographic information (gender, age), comorbidities (acute kidney failure, atrial fibrillation, chronic kidney disease, diabetes), medication during hospitalization (acetaminophen, heparin, morphine), laboratory indicators (base excess, calculate total CO2, chloride, free calcium, glucose, lactate, pCO2, pH, pO2, potassium, sodium, basophils, neutrophils, albumin, anion gap, bicarbonate, calcium, creatinine, chloride, urea nitrogen, hemoglobin, platelets, hematocrits, oxygen saturated hemoglobin, prothrombin time, partial thromboplastin time, mean corpuscular hemoglobin, mean corpuscular hemoglobin concentration, red blood cell, red cell distribution width, white blood cell, alanine aminotransferase, aspartate aminotransferase, total bilirubin, creatine kinase isoenzyme MB, and lactate dehydrogenase). Diagnosis of IS, infarction location, and complications were based on the international classification of diseases, ninth edition (ICD‐9) and tenth edition (ICD‐10). For multiple measurements, we used data within 24 h of admission. If multiple data were available within 24, the first measurement was analyzed. The starting point of follow‐up was the time the patient was admitted to the hospital, and the end point of follow‐up was the time the patient developed respiratory failure or was discharged from the hospital. It was confirmed that data of each patient were produced during one admission through the patients` hospitalization number (HADM_ID) provided in the database.

### 2.2 Statistical Analysis

The study cohort was divided in a 7:3 ratio, into a training set and a validation set. Frequency and percentile were used to express categorical variables (%), and differences between the groups were analyzed using the chi‐square test. A normality test was performed for continuous variables. Un‐normally distributed continuous variables were expressed using median with quartiles, and differences between the groups were analyzed using the rank‐sum test. Normally distributed continuous variables were expressed by the mean with standard deviation, and differences between the groups were assessed using a *t*‐test. Univariate Cox regression and akaike information criterion (AIC)‐based two‐way stepwise Cox regression were applied in the training set to select variables. Variables of statistical significance in univariate regression were included in Cox stepwise regression, and those of statistical significance in stepwise regression were finally included in the nomogram. To avoid multicollinearity, we calculated the variance inflation factor (VIF) for each variable in the model, and variables with VIF > 5 were excluded. For the variables in the model, we used MSCM to adjust for confounders such as baseline and time‐varying variables for causal inference. MSCM is a statistical model for causal inference based on inverse probability weighting. This model combines the features of the traditional Cox proportional risk model and the MSCM and is used to address the time‐dependent bias that can occur in traditional regression models when dealing with long‐term or repeated interventions (Robins et al. 2000). In addition, the receiver operating characteristic curve (ROC) was applied and the area under the curve (AUC) was calculated to assess the accuracy of the model. The calibration curve was used to assess the consistency between predicted values, and the actual ones. Data analyses were performed using R software (ver. 4.2.1, https://www.r‐project.org/). A *p*‐value less than 0.05 indicated statistical significance. The rms package (ver.6.3.0) of R was used for plotting the nomogram and calibration curve, survival (ver.3.3.1) was for Cox regression, step function for variable‐selection in stepwise Cox regression, and survival ROC (ver.1.0.3) for plotting ROC.

## 3 Results

**FIGURE 1:**
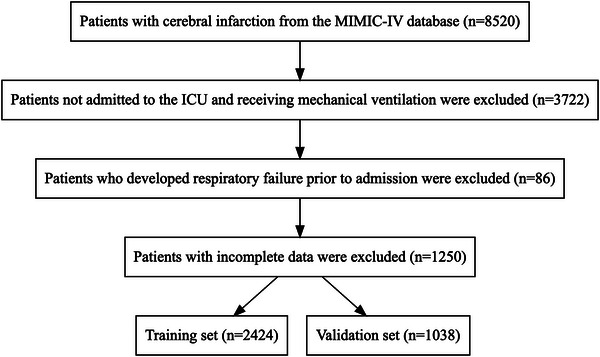
Flow chart of patient selection.

Data of 8520 IS patients were identified from the MIMIC‐IV 2.0 database (ICD 9: 433, 434; ICD 10: I63), and 5058 patients were excluded due to incomplete data and respiratory failure onset before ICU admission, baseline characteristics of excluded patients are shown in Supplementary Table 1 (Supplementary document 1). A total of 3462 patients were finally included in this study (Figure 1). Respiratory failure occurred in 43 patients (1.2%). The median follow‐up duration was 5.38 days. Among the 2424 patients in the training set, 32 had nosocomial respiratory failure, with a median follow‐up duration of 5.67 days. Among the 1038 patients in the validation set, 11 had nosocomial respiratory failure, with a median follow‐up duration of 5.04 days. There was no statistical difference in the incidence of respiratory failure (*p* = 0.641) and in follow‐up duration (*p* = 0.364) between the two sets, indicating comparability of the data. The median age of the cohort was 71.00 (61.00, 80.00) years, and the male patients (54.4%) slightly outnumbered the female patients (45.6%). Details of the data are shown in Table 1. A total of 113 patients with IS in the eICU were included in the study for external validation. Patient characteristics are shown in Supplementary Table 2 (Supplementary document 1).

**TABLE 1:** Characteristics of all patients.

—	Overall	Training set	Validation set	*p* value
*n*	3462	2424	1038	—
RF (%)	43 (1.2)	32 (1.3)	11 (1.1)	0.641
Time (Days)	5.38 [2.09, 11.13]	5.67 [2.09, 11.67]	5.04 [2.12, 10.63]	0.364
Gender = Male (%)	1883 (54.4)	1319 (54.4)	564 (54.3)	0.996
Age (Years old)	71.00 [61.00, 80.00]	71.00 [61.00, 79.00]	72.00 [62.00, 80.00]	0.016
Infarction location (%)	—	—	—	0.987
Basilar artery	59 (1.7)	39 (1.6)	20 (1.9)	—
Carotid artery	1003 (29.0)	706 (29.1)	297 (28.6)	—
Cerebellar artery	50 (1.4)	37 (1.5)	13 (1.3)	—
Mesencephalic arteries	219 (6.3)	155 (6.4)	64 (6.2)	—
Another anterior cerebral artery	242 (7.0)	166 (6.8)	76 (7.3)	—
Posterior cerebral artery	51 (1.5)	37 (1.5)	14 (1.3)	—
Unspecified	1772 (51.2)	1238 (51.1)	534 (51.4)	—
Vertebral artery	66 (1.9)	46 (1.9)	20 (1.9)	—
AKI = Yes (%)	1553 (44.9)	1091 (45.0)	462 (44.5)	0.815
AF = Yes (%)	1167 (33.7)	801 (33.0)	366 (35.3)	0.221
Diabetes = Yes (%)	1503 (43.4)	1076 (44.4)	427 (41.1)	0.083
COPD = Yes (%)	905 (26.1)	638 (26.3)	267 (25.7)	0.746
CKD = Yes (%)	1192 (34.4)	862 (35.6)	330 (31.8)	0.036
Acetaminophen = Yes (%)	102 (2.9)	70 (2.9)	32 (3.1)	0.84
Heparin = Yes (%)	112 (3.2)	81 (3.3)	31 (3.0)	0.663
Morphine = Yes (%)	44 (1.3)	31 (1.3)	13 (1.3)	1
Base excess, mEq/L	0.00 [–2.00, 2.00]	0.00 [–1.00, 2.00]	0.00 [–2.00, 2.00]	0.212
Calculate total CO2, mEq/L	25.00 [23.00, 28.00]	25.00 [23.00, 28.00]	25.00 [23.00, 28.00]	0.098
Chloride, mEq/L	103.53 [101.00, 106.00]	103.32 [101.00, 106.00]	103.93 [101.00, 106.00]	0.111
Free calcium, mmol/L	1.13 [1.10, 1.16]	1.13 [1.10, 1.16]	1.13 [1.10, 1.16]	0.963
Glucose, mg/dL	129.80 [107.00, 154.07]	130.50 [109.00, 155.00]	127.00 [105.00, 151.00]	0.004
HCT, %	34.12 [31.00, 38.44]	34.00 [30.48, 38.15]	34.94 [31.00, 38.91]	0.251
Lactate, mmol/L	1.50 [1.12, 1.90]	1.50 [1.10, 1.89]	1.50 [1.20, 2.00]	0.001
O2 SAT, %	92.00 [87.00, 97.00]	91.87 [86.74, 97.00]	92.22 [87.66, 97.00]	0.038
pCO2, mm Hg	39.00 [35.00, 44.00]	39.00 [35.00, 44.00]	39.00 [35.00, 44.00]	0.974
pH	7.40 [7.36, 7.44]	7.40 [7.36, 7.44]	7.40 [7.36, 7.44]	0.63
pO2, mm Hg	168.00 [93.00, 314.00]	165.50 [92.00, 310.25]	177.50 [96.00, 318.75]	0.198
Potassium, mEq/L	4.07 [3.80, 4.40]	4.08 [3.80, 4.40]	4.04 [3.80, 4.32]	0.407
Sodium, mEq/L	138.00 [136.00, 139.19]	138.00 [136.00, 139.19]	138.00 [136.00, 139.15]	0.624
Basophils, %	0.40 [0.20, 0.60]	0.40 [0.20, 0.60]	0.40 [0.20, 0.60]	0.836
Neutrophils, %	73.00 [64.90, 81.97]	73.40 [65.30, 82.00]	72.00 [63.70, 81.68]	0.025
Albumin, g/dL	3.80 [3.40, 4.20]	3.80 [3.40, 4.20]	3.80 [3.40, 4.20]	0.846
Anion gap, mEq/L	15.00 [13.00, 17.00]	15.00 [13.00, 17.00]	15.00 [13.00, 17.00]	0.258
Bicarbonate, mEq/L	26.00 [23.00, 28.00]	26.00 [23.00, 28.00]	26.00 [23.00, 28.00]	0.618
Calcium, mg/dL	8.90 [8.40, 9.40]	8.90 [8.40, 9.40]	8.90 [8.30, 9.30]	0.537
Creatinine, mg/dL	1.00 [0.80, 1.30]	1.00 [0.80, 1.30]	1.00 [0.80, 1.30]	0.129
Urea nitrogen, mg/dL	20.00 [15.00, 27.00]	20.00 [15.00, 28.00]	19.00 [15.00, 27.00]	0.488
INR	1.10 [1.00, 1.20]	1.10 [1.00, 1.20]	1.10 [1.00, 1.20]	0.458
PT, sec	12.60 [11.60, 14.00]	12.60 [11.60, 14.00]	12.40 [11.50, 13.80]	0.3
PTT, sec	28.80 [25.70, 32.50]	28.70 [25.80, 32.60]	28.80 [25.60, 32.20]	0.302
Hemoglobin, g/dL	12.70 [11.40, 14.10]	12.70 [11.30, 14.00]	12.90 [11.40, 14.20]	0.061
MCH, pg	30.40 [28.90, 31.70]	30.30 [28.90, 31.70]	30.50 [29.10, 31.80]	0.036
MCHC, %	33.40 [32.50, 34.40]	33.40 [32.50, 34.30]	33.45 [32.50, 34.50]	0.324
MCV, fL	90.00 [87.00, 94.00]	90.00 [87.00, 94.00]	91.00 [87.00, 95.00]	0.146
Platelets, K/uL	233.00 [186.00, 289.00]	232.00 [185.00, 286.00]	235.00 [188.00, 293.00]	0.113
RBC, m/uL	4.23 [3.75, 4.66]	4.22 [3.75, 4.65]	4.26 [3.76, 4.68]	0.221
RDW, %	13.80 [13.20, 14.80]	13.90 [13.20, 14.80]	13.80 [13.20, 14.70]	0.06
WBC, K/uL	8.45 [6.60, 11.10]	8.50 [6.60, 11.10]	8.30 [6.60, 11.00]	0.513
ALT, IU/L	22.00 [15.00, 33.00]	22.00 [15.00, 33.34]	22.00 [15.00, 32.00]	0.622
ALP, IU/L	77.00 [62.00, 96.00]	77.01 [62.00, 97.00]	76.00 [61.00, 93.79]	0.048
AST, IU/L	25.00 [20.00, 36.16]	25.00 [20.00, 37.00]	25.00 [19.00, 35.00]	0.404
TBIL, mg/dL	0.50 [0.30, 0.70]	0.50 [0.30, 0.70]	0.50 [0.30, 0.70]	0.603
CKMB, ng/mL	4.42 [3.00, 7.85]	4.40 [3.00, 7.91]	4.50 [3.00, 7.38]	0.54
LDH, IU/L	235.06 [198.00, 297.14]	236.00 [199.00, 300.00]	234.00 [197.00, 286.75]	0.166

Abbreviations: AKI: acute kidney failure; AF: Atrial Fibrillation; CKD: chronic kidney disease; HCT: hematocrits; O2 SAT: oxygen saturated hemoglobin; PT: prothrombin time; PTT: partial thromboplastin time; MCH: mean corpuscular hemoglobin; MCHC: mean corpuscular hemoglobin concentration; RBC: red blood cell; RDW: red cell distribution width; WBC: white blood cell; ALT: alanine aminotransferase; AST: aspartate aminotransferase; TBIL: total bilirubin; CKMB: Creatine kinase isoenzyme MB; LDH: Lactate dehydrogenase.

### 3.1 Model Construction and Validation

**FIGURE 2:**
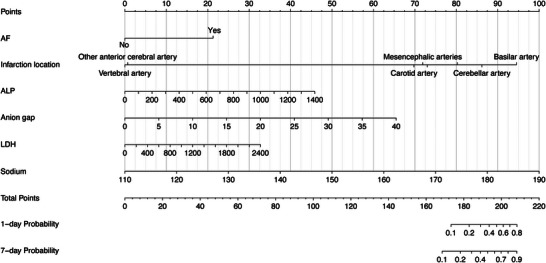
The nomogram of the Cox proportional hazard ratio model. The final risk score was calculated by adding up the score of each item.

**FIGURE 3:**
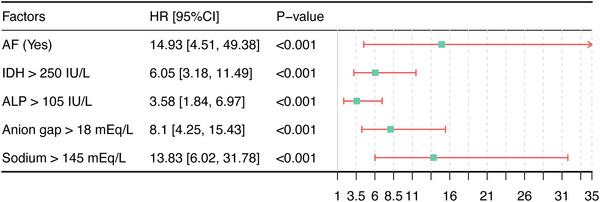
Results of MSCM analysis.

Univariate regression showed that infarction location, atrial fibrillation, alkaline phosphatase (ALP), anion Gap (AG), aspartate aminotransferase (AST), serum creatinine (SCr), blood glucose, lactic acid, lactic dehydrogenase (LDH), pH, pO2, red blood cell distribution width (RDW), sodium ion, and blood urea nitrogen (BUN) were significantly associated with the occurrence of respiratory failure in IS patients. These variables were included in stepwise Cox regression for further screening, and the results showed that atrial fibrillation (HR [95%CI]: 5.25 [1.22–22.52], *p* = 0.0257*), ALP (HR [95%CI]: 1.003 [1–1.005], *p* = 0.0160), AG (HR [95%CI]: 1.14 [1.05–1.23], *p* = 0.0011), LDH (HR [95%CI]: 1.001 [1.000–1.002], *p* = 0.0172), and sodium ion (HR [95%CI]: 1.1 [1.06–1.15], *p* < 0.0001) were independent predictors for the occurrence of respiratory failure in IS patients (Table 2). Cox proportional hazard model was constructed based on the above variables, and a nomogram was plotted as shown in Figure 2. In the nomogram, the total score (total Points) was determined by summing the scores (Points) of each variable. The total score (Total Points) corresponded vertically to the scale of the predictor below (1‐day Probability, 7‐day Probability, i.e. the patient's risk of experiencing respiratory failure. For the laboratory indicators in the model, we used the reference ranges provided by the MIMIC database as a grouping of cutoff values for inclusion in the MSCM analysis. We did not include infarction location in the MSCM analysis because of the large number of subcategories of infarction location and the lack of an appropriate grouping strategy. The MSCM results are consistent with the model (Figure 3).

**TABLE 2:** Feature selection results of univariate and multivariate analyses.

Factors	Levels	Univariate analysis		Multivariate analysis	
		HR (95% CI)	*p* value	HR (95% CI)	*p* value
Acetaminophen	No (ref)	—	—	—	—
—	Yes	1.08 (0.15–7.89)	0.9417	5.25 (1.22–22.52)	0.0257
AF	No (ref)	—	—	—	—
—	Yes	5.62 (1.34–23.53)	0.0181*	5.331 (1.269–22.400)	0.0223*
AKI	No (Ref)	—	—	—	—
—	Yes	2.19 (0.3–16.06)	0.4398	—	—
Infarction location	Basilar artery (Ref)	—	—	—	—
—	Carotid artery	0.11 (0.03‐0.44)	0.0019	0.15 (0.04–0.59)	0.0066
—	Cerebellar artery	0.34 (0.04–3.29)	0.3537	0.52 (0.05–5.1)	0.5767
—	Mesencephalic arteries	0.16 (0.03–0.96)	0.0455	0.17 (0.03–1.05)	0.056
—	Another anterior cerebral artery	0 (0–Inf)	0.9967	0 (0–Inf)	0.9969
—	Posterior cerebral artery	0.34 (0.04–3.27)	0.3505	0.33 (0.03–3.25)	0.3412
—	Unspecified	0.19 (0.06–0.66)	0.0083	0.19 (0.05–0.65)	0.0083
—	Vertebral artery	0 (0–Inf)	0.9983	0 (0–Inf)	0.9984
Albumin	—	0.65 (0.39–1.08)	0.0948	—	—
ALP	—	1.00 (1.00–1.00)	0.0106*	1.003 (1–1.005)	0.0160*
ALT	—	1.00 (1.00–1.00)	0.1333	—	—
Age	—	1.00 (0.97–1.02)	0.9342	—	—
Anion gap	—	1.16 (1.08–1.24)	<0.0001*	1.14 (1.05–1.23)	0.0011*
AST	—	1.00 (1.00–1.00)	0.0253*	—	—
Base excess	—	0.97 (0.9–1.04)	0.3598	—	—
Basophils	—	1.25 (0.7–2.24)	0.4589	—	—
Bicarbonate	—	0.95 (0.87–1.04)	0.2752	—	—
Calcium	—	0.71 (0.46–1.12)	0.1418	—	—
Calculate total CO2	—	1.06 (0.97–1.15)	0.2211	—	—
Chloride	—	1.01 (0.94–1.09)	0.7606	—	—
Chloride	—	1.01 (0.94–1.09)	0.7595	—	—
CKD	—	3.61 (0.86–15.13)	0.0785	—	—
CKMB	—	1.00 (0.99–1.01)	0.7910	—	—
COPD	No (ref)	—	—	—	—
—	Yes	1.31 (0.62–2.76)	0.4811	—	—
Creatinine	—	1.20 (1.01–1.43)	0.0424*	—	—
Diabetes	—	3.31 (0.79–13.84)	0.1015	—	—
Free calcium	—	0.04 (0.00–17.85)	0.2961	—	—
Gender	Female (ref)	—	—	—	—
—	Male	1.23 (0.61–2.49)	0.5621	—	—
Glucose	—	1.01 (1.00–1.01)	0.0081*	—	—
HCT	—	1.00 (0.94–1.06)	0.9266	—	—
Hemoglobin	—	0.90 (0.77–1.07)	0.2295	—	—
Heparin	—	0.91 (0.12–6.66)	0.9257	—	—
INR	—	1.25 (0.8–1.96)	0.3284	—	—
Lactate	—	1.73 (1.1–2.73)	0.0185*	—	—
LDH	—	1.00 (1.00–1.00)	0.0009*	1.001 (1–1.002)	0.0172*
MCH	—	1.02 (0.89–1.18)	0.7597	—	—
MCHC	—	0.82 (0.66–1.00)	0.0527	—	—
MCV	—	1.04 (0.99–1.10)	0.1043	—	—
Morphine	No (ref)	—		—	—
—	Yes	2.57 (0.35–18.85)	0.3523	—	—
Neutrophils	—	1.03 (1.00–1.06)	0.0792	—	—
O2 SAT	—	1.00 (0.97–1.04)	0.7949	—	—
pCO2	—	1.04 (0.99–1.09)	0.1090	—	—
pH	—	0.00 (0.00–0.86)	0.0445*	—	—
Platelets	—	1.00 (1.00–1.00)	0.5890	—	—
pO2	—	1.00 (0.99–1.00)	0.0361*	—	—
Potassium	—	1.65 (0.91–2.99)	0.0997	—	—
PT	—	1.02 (0.97–1.07)	0.4119	—	—
PTT	—	1.00 (0.97–1.03)	0.9848	—	—
RBC	—	0.74 (0.44–1.22)	0.2331	—	—
RDW	—	1.18 (1.01–1.37)	0.0415*	—	—
Sodium	—	1.10 (1.05–1.14)	<0.0001*	1.1 (1.06–1.15)	< 0.0001*
TBIL	—	1.06 (0.90–1.25)	0.4962	—	—
Urea nitrogen	—	1.02 (1.00–1.03)	0.0249*	—	—
WBC	—	1.04 (0.99–1.09)	0.1244	—	—

*Note*: *: *p* value < 0.05.

**FIGURE 4:**
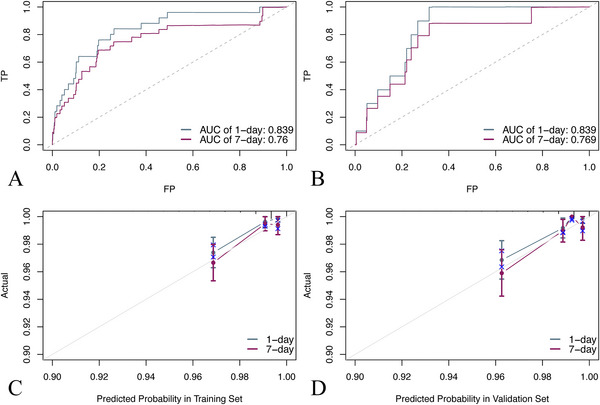
(A) The ROC curve of predicting respiratory failure in one and seven days in the training cohort; (B) The ROC curve of predicting respiratory failure in one and seven days in the validation cohort; (C, D) The calibration curve of predicting respiratory failure in one and seven days in the training cohort and validation cohort, with the nomogram, predicted probability of survival shown on the x‐axis and actual survival proportion shown on the y‐axis. Error bars represent 95% confidence intervals.

**FIGURE 5:**
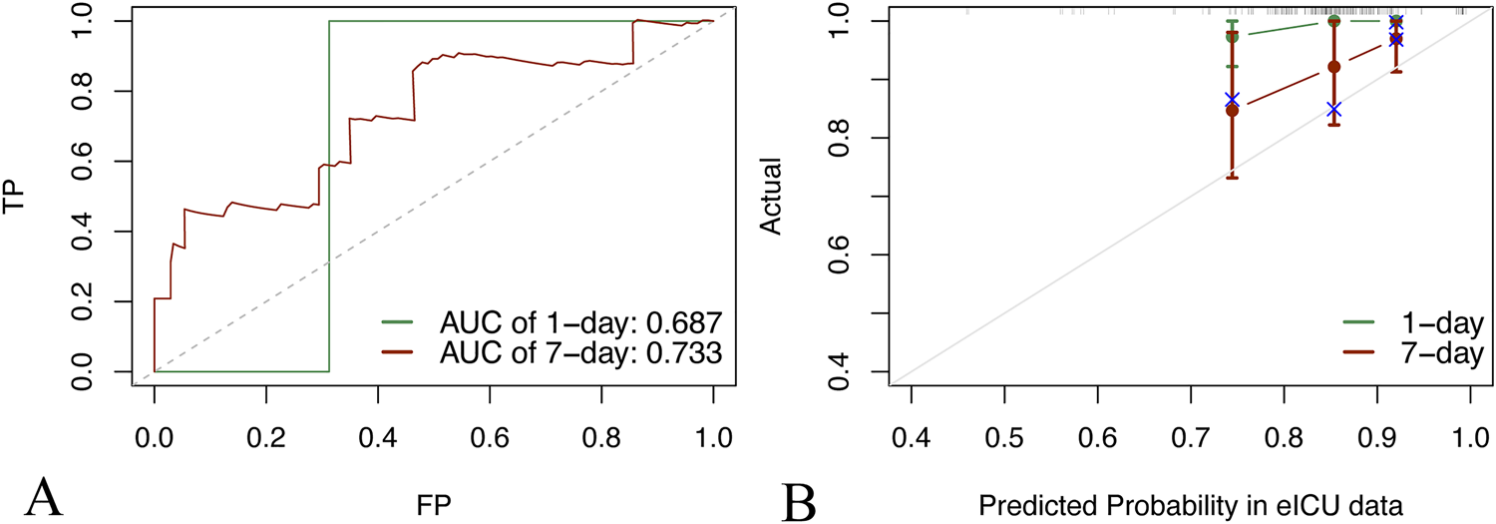
Results of external validation. (A) The ROC curve of predicting respiratory failure in one and seven days in the eICU cohort. (B) The calibration curve of predicting respiratory failure in one and seven days in the eICU cohort.

ROC and calibration curves were provided to validate the model. The results showed that the AUC of respiratory failure onset within 1 and 7 days after admission was 0.839 and 0.760 in the training set (Figure 4 A), 0.839 and 0.769 in the validation set (Figure 4B), and 0.687 and 0.733 in the eICU set (Figure 5A), respectively. The model presented to be of considerable recognizing ability. The calibration curve showed that the model neither significantly overestimated nor underestimated the risk (Figure 4C, 4D, 5B).

**FIGURE 6:**
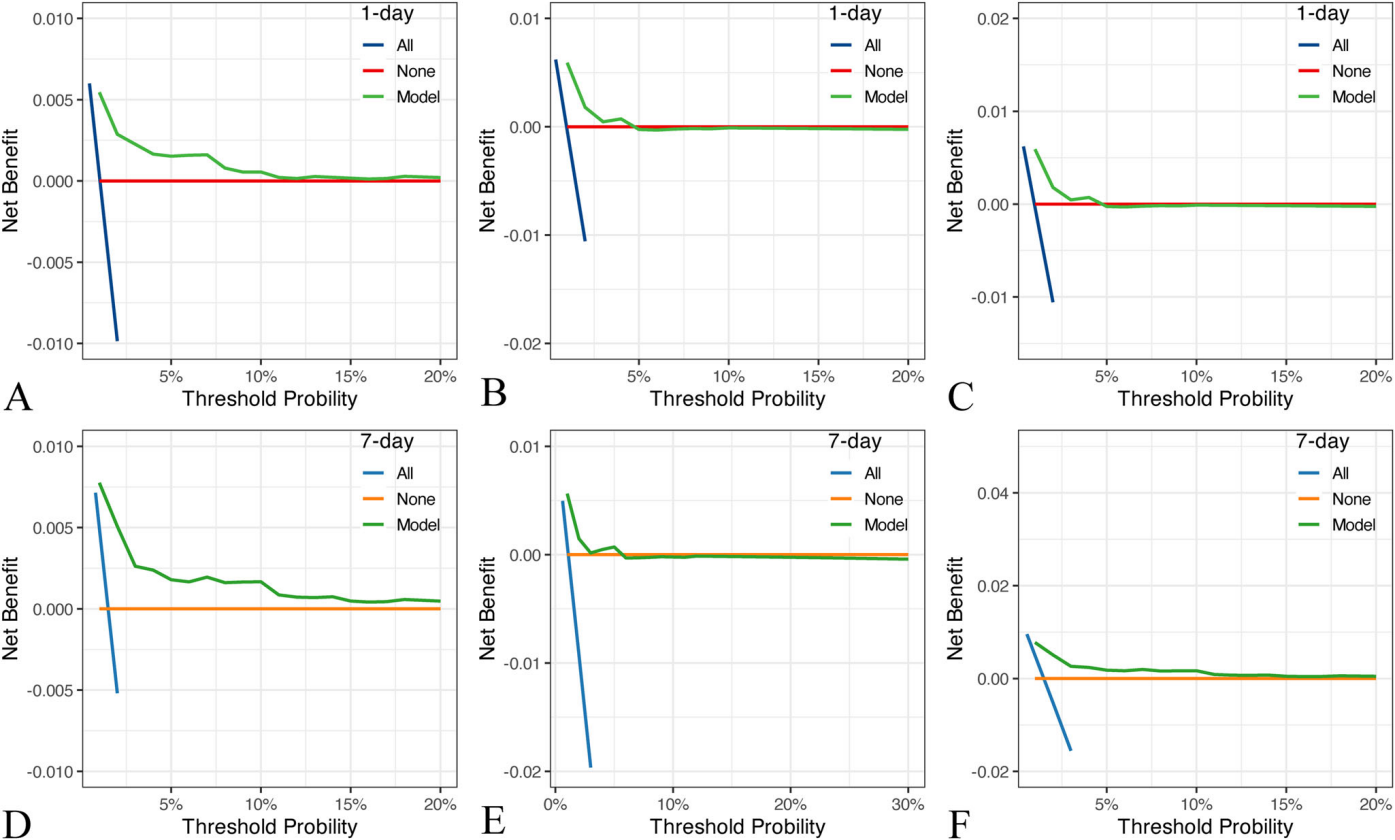
Results of decision curve analysis. (A, B, C) The decision curves of predicting respiratory failure in one day in the training, validation and eICU cohort. (D, E, F) the decision curves of predicting respiratory failure in seven days in the training, validation and eICU cohort.

Finally, we performed decision curve analysis in the training set, the validation set and the eICU set. The results showed that the model has some net benefit in all three datasets (Figure 6).

## 4 Discussion

In this study, we have used a large number of ICU‐admitted IS cases to construct and validate a nomogram model for the prediction of respiratory failure, which includes five independent predictors based on conventional and available demographic information, laboratory indicators, comorbidities, and medication history. The model presents to be practical. Clinicians could be able to identify patients with a high risk for respiratory failure with the assistance of this model, so that they could take timely intervention.

Our analysis showed that infarction location, atrial fibrillation, ALP, AG, LDH, and sodium ion were independent predictors for the in‐hospital onset of respiratory failure in patients with IS. Atrial fibrillation is a major risk factor for acute stroke. Studies indicate that cardioembolic stroke is one of the primary types of acute stroke, accounting for approximately 20% of all acute stroke cases (and up to 25% of ischemic cerebrovascular events). Among arrhythmia‐related stroke etiologies, atrial fibrillation accounts for 37% (Capmany et al. 2004). We presume that the first measurement of pO2 or pCO2 after admission would be a significant predictor for respiratory failure. However, the results indicate statistical significance of pO2 only in univariate regression, and the HR is close to 1. In multivariate analysis that contained other factors, pO2 showed no significance. There is no statistical significance of pCO2 in both univariate and multivariate regression.

Our study shows that cerebral infarction is less likely to occur in the carotid artery compared to the basilar artery for RF (OR = 0.15, 95%CI = 0.04–0.59, *p* = 0.0066). The carotid and basilar arteries are both important blood vessels in the body but differ in their location to the respiratory center. The basilar artery is located at the base of the brain and is responsible for supplying the deeper structures of the brain, such as the brainstem, thalamus, and subcortical areas of the brain. The respiratory center, on the other hand, is located primarily in the medulla oblongata portion of the brainstem and controls the rhythm and depth of breathing. Therefore, the basilar artery is closer relative to the respiratory center, and it is reasonable to assume that infarction of the basilar artery is more likely to affect respiratory function (Guyenet and Bayliss 2015). Furthermore, our study has also shown that concomitance with atrial fibrillation and increased ALP, AG, LDH, and Na^2+^ are independent risk factors for respiratory failure in IS patients. A consensus has been reached that cardiovascular diseases such as atrial fibrillation could induce respiratory disturbance (Somers et al. 2008). ALP typically serves as a marker for liver dysfunction (Suzuki et al. 2006, Salahshoor et al. 2018, Zhong et al. 2021, Stirnadel‐Farrant et al. 2015), as well as a phenotypic marker that reflects osteoblast activity (Magnusson et al. 2002, Hartwell et al. 2007, Nizet et al. 2020, Sardiwal et al. 2013). However, we have observed that increased ALP is significantly associated with respiratory failure in IS patients, which could be attributed to the decreased hepatic immunity leading to an increased risk of upper respiratory infection (Kubes and Jenne 2018). AG and Na^2+^ concentrations usually reveal acid‐base and electrolyte balance in the internal environment (Langman et al. 2019, Moe and Fuster 2003). Severe hypoxemia and shock induce hypoxia and massive lactic acid generation, and those acid metabolites could decrease serum HCO3^−^, leading to an increase in AG (Kraut and Madias 2014, Fenves and Emmett 2021, Xu et al. 2017). On the other hand, two studies have found an association between AG and adverse neuropath logical consequences. The study by Hesong Wang et al. indicates that higher AG concentration is significantly associated with increased short‐term mortality and all‐cause mortality in IS patients receiving rtPA treatment (Wang et al. 2022). Another study suggests that AG would be a potential marker for the long‐term prognosis of patients with spontaneous cerebral hemorrhage, and rectification of AG could improve the patients` prognosis (Shen et al. 2022). As for Na^2+^ concentration, there is no study that provides a direct discussion of the association between hypernatremia and impaired respiratory function, while studies indicate hypernatremia could increase the mortality of several respiratory diseases (Tzoulis et al. 2021). Our study has also shown that increased LDH is a potential predictor for respiratory failure. Lactate dehydrogenase catalyzes the mutual conversion of pyruvate and lactic acid. Hypoxia or insufficient oxygen supply converts pyruvate, the final product of glycolysis, into lactic acid. To sum up, these biomarkers may be associated with the biochemical reaction in IS patients before the occurrence of respiratory failure and can be used as an effective predictor for respiratory failure in IS patients.

Before this study, there was no prediction model developed to predict the risk of respiratory failure in IS patients admitted to ICU, or to assess the association between IS and respiratory failure. Sleep‐related breathing disorders are also a significant cause of acute stroke (Uscamaita et al. 2025). Several studies have provided prediction models for sleep‐related respiration disorder in IS patients, among which one study involving 120 acute IS patients has yielded an AUC of up to 0.81 (Siarnik et al. 2021), and this model indicates that body mass index (BMI), stroke after awakening, and diastolic dysfunction are independently associated with the occurrence of sleep‐related respiration disorder. Another study is based on 1330 stroke patients, and has applied random forest for modeling, with the AUC of up to 0.75 (Brown et al. 2020). However, the study did not account for follow‐up time. Our model has narrowed the study to patients with IS in the ICU, who are more severe and more urgent for respiratory intervention. In addition, we selected respiratory failure as our outcome indicator and took into account the time of the onset. The constructed nomogram can quantify the individual risk of patients, and the AUC is more than 0.8. The model can provide reliable predictive information for clinicians. There are, however, some limitations of this study that must be mentioned: first, in this study, patients with less than one day of follow‐up were excluded. Second, the dataset used for external validation in this study is small, and a larger sample is needed for validation. Additionally, we were unable to observe respiratory failure in patients who had been discharged from the hospital. Consequently, we observed a low incidence of respiratory failure in the study population. Furthermore, due to the limitations of the database, we were unable to include certain indicators of interest, such as brain imaging data from patients. Finally, this study employed a retrospective design, and its conclusions relied on existing data within the database. Due to missing information on the critical variable (cause of death), we were unable to conduct a relevant analysis.

## 5 Conclusion

We have developed a nomogram for predicting respiratory failure in IS patients admitted to the ICU, which considers the time of disease onset. The nomogram indicates that concomitance with atrial fibrillation and increased ALP, AG, LDH, and atrial fibrillation could increase the risk for respiratory failure in IS patients. This model could provide useful predictive information to help clinicians identify high‐risk patients. Future studies should independently validate the predictive efficacy of this model in large‐scale prospective cohorts. More efforts should be made to systematical elucidate the pathophysiological mechanisms underlying post‐stroke respiratory failure, including damage to the respiratory center, neurogenic respiratory muscle dysfunction, and sleep‐related breathing disorders.

## Author Contributions

**Zhenjun Liu**: Conceptualization, Methodology, Formal analysis and investigation, Writing – original draft preparation, Writing – review and editing, Resources. **Luolan Gui**: Conceptualization, Methodology, Formal analysis and investigation. **Qian Zhao**: Writing – original draft preparation. **Yi L**: Writing – review and editing, Funding acquisition, Resources, Supervision

## Funding

The work was supported by the Key Clinical Construction Project of Sichuan Province.

## Ethics Statement

The authors have nothing to report.

## Conflicts of Interest

The authors declare no conflicts of interests.

## Supporting information




**Supplementary Material**: brb371146‐supp‐0001‐TableS1‐S2

## Data Availability

Data of IS patients in the MIMIC‐IV database (ver.2.0, available from: https://physionet.org/content/mimiciv/2.0/) and eICU database (ver.2.0, https://physionet.org/content/eicu‐crd/2.0/)
